# The use of newly isolated fungal cultures for the selective delignification of bamboo culms

**DOI:** 10.3389/fbioe.2023.1265420

**Published:** 2023-08-31

**Authors:** Bo Zhao, Rui Liu, Qi Guo, Gang Xu, Li Zhang, Peng Sun, Ying Cao, Shanglian Hu

**Affiliations:** ^1^ School of Life Science and Engineering, Bamboo Research Institute, Southwest University of Science and Technology, Mianyang, China; ^2^ Sichuan Academy of Forestry, Chengdu, China

**Keywords:** combined fungi, ligninolytic enzyme production, bamboo, selective degradation, orthogonal test

## Abstract

The screening of ligninolytic enzyme-producing fungal species in samples led to the identification of *Paracremonium* sp. LCB1, *Clonostachys compactiuscula* LCD1 and *C. compactiuscula* LCN1. Both these strains produced high levels of hemicellulase and ligninolytic enzyme production over a relatively short fermentation period of 3–5 days while exhibiting very low levels of cellulase activity. The results of the tests indicated that co-culturing LCB1 and LCN1 enhanced the ability to degrade lignin, and the ideal degrading circumstances and internal degrading mechanism of combined fungi were examined. The results showed that under conditions of temperature (30°C), pH (5), culture time (40 d), solid-liquid ratio (1:2.5), the pretreatment of bamboo culms with a co-culture of LCB1 and LCN1 resulted in a pronounced 76.37% drop in lignin weight and a high lignin/cellulose loss ratio (>10). Fourier transform infrared spectroscopy, X-ray diffractometry, and scanning electron microscopy were used to characterize the physicochemical properties of these bio-pretreated bamboo culms, further confirming that LCB1 and LCN1 co-culture represents an effective approach to bamboo delignification.

## 1 Introduction

Owing to its rapid growth, ease of propagation, and convenience for harvesting, bamboo is among the most valuable feedstocks as a source of crop residues. Bamboo residues consist of high levels of cellulose, hemicellulose, and lignin, the former two of which offer a high degree of value in the context of fermentable sugar production (Lin et al., 2019). However, pretreatment is necessary to degrade lignin within bamboo culms prior to their use for energy production given that the lignin network will otherwise interfere with the efficacy of enzymatic treatment and the consequent release of fermentable sugars. While various thermochemical pretreatment protocols have been developed to reduce the adverse effects of lignin, these protocols are generally energy-intensive, harsh, and environmentally damaging such that they produce toxic byproducts which can interfere with subsequent microbial fermentation (Ma et al., 2017). In some reports, biological pretreatment has been reported to enhance lignocellulose degradation while offering key advantages including lower costs, milder pretreatment conditions, and the absence of any associated environmental pollution (Lubbers et al., 2019; Shi et al., 2019). Fungi generally outperform bacteria in the context of lignin degradation, as they exhibit greater accessibility and can produce higher levels of ligninolytic enzymes including lignin peroxidase (LiP), manganese-dependent peroxidase (MnP), and laccase (Lac). LiP and MnP catalyze the oxidation of nonphenolic and phenolic substrates with H_2_O_2_ as the oxidant and have high redox potentials that allow oxidation of recalcitrant lignin. Lac acts along with lignin peroxidase and manganese peroxidase leading to complete degradation of lignin (Voberkova et al., 2018).

Selective lignin-degrading fungi are particularly promising candidates for use in bamboo bio-pretreatment (Suhara et al., 2012; Ma et al., 2017; Xue et al., 2022). Accordingly, there have been many published studies focused on screening for fungal strains capable of degrading bamboo-derived lignin and optimizing these fungal delignification conditions have been published (Zhang et al., 2007; Suhara et al., 2012; Zhang et al., 2017). However, there remains a pressing lack of fungal species capable of preferentially degrading lignin while leaving cellulose intact for subsequent saccharification. Individual microbial treatments are generally time-intensive and exhibit relatively low levels of bioconversion efficacy. However, pretreatment with a synergistic fungal co-culture may represent a more efficient approach to lignin depolymerization. Indeed, in prior studies, combining two to three fungi has been demonstrated to markedly improve lignin depolymerization as compared to the use of a single fungus (Ma and Ruan, 2015; Meehnian et al., 2017).

While screening for lignin-degrading enzyme producers among isolates from Longchang (Sichuan Province, China), the *Paracremonium* sp. Strain LCB1, *Clonostachys compactiuscula* strains LCD1 and LCN1 were found to exhibit notably higher levels of ligninolytic activity than any other tested strains. Accordingly, this study was developed to explore the lignin-degrading activity of these strains, ultimately revealing that LCB1 and LCN1 hold great promise as fungi that can be employed for on-site ligninolytic enzyme production and bamboo delignification. The ideal culture conditions for lignin breakdown were analyzed and determined by combining single-factor test and an orthogonal test.

## 2 Materials and methods

### 2.1 Raw materials

*Dendrocalamus latiflorus* Munro, commonly referred to as ma bamboo, was kindly provided by scientific research base of Sichuan Academy of Forestry (Longchang, China). To prepare samples for processing, bamboo culms were crushed through a 0.9 mm sieve and dried for 3 days at 60°C prior to use.

### 2.2 Fungal culture

Pure cultures of the LCB1, LCD1 and LCN1 strains were maintained in our laboratory. These fungi were maintained on potato dextrose agar (PDA) slants at 4°C. To prepare inoculums for fungal pretreatment, these cultures were grown for 10 days on PDA plates at 28°C. The initial medium prepared to analyze enzyme production consisted of 0.2% (w/v) (NH_4_)_2_SO_4_, 0.2% (w/v) K_2_HPO_4_, 0.03% (w/v) MgSO_4_·7H_2_O, and 2% (w/v) bamboo powder, with a pH that was adjusted to 7.0.

### 2.3 Analyses of enzymatic activity

LCB1, LCD1 and LCN1 strains were initially cultured in the prepared enzyme production medium for 6 days, with 1.5 mL aliquots being collected daily. These samples were centrifuged for 10 min at 10,000 rpm, and the filter paper activity (FPase) and xylanase activity levels in the supernatant fraction were then measured as reported previously (Meng et al., 2018). Supernatant Lac, LiP, and MnP activity levels were measured with an enzyme activity assay kit (Solarbio, Beijing, China). Three biological replicate samples were used when conducting all experiments.

### 2.4 Fungal pretreatment

A 250 mL Erlenmeyer flask containing 100 mL of PDB medium (pH 5.5) was inoculated with 3-5 disks collected from the edge of active fungal cultures grown on PDA plates. These flasks were then incubated for 3 days at 28°C with constant agitation (180 rpm) to prepare seed cultures. Fungal pretreatment was performed in 250 mL Erlenmeyer flasks containing 10 g of pulverized bamboo in a certain amount of dH_2_O that had been sterilized (30 min, 121°C) followed by aseptic inoculation with 5 mL of fungal seed cultures for the single culture groups, or 2.5 mL each of the seed cultures for the two fungi co-culture treatment group, or 1.7 mL each of the seed cultures for the three fungi co-culture treatment group. Non-inoculated control samples were incubated under these same conditions. All flasks were incubated for 30–50 days, 28°C–32°C, and pH 5-7 under static conditions, dried for 3 days at 60°C, and used for physicochemical and structural characterization. All cultures were prepared in triplicate.

### 2.5 Single factor test and orthogonal test

As shown in Table 1, four factors–temperature, pH, culture period, and solid-liquid ratio–were carefully chosen based on the findings of the pre-test. Three levels were employed for each factor. The weight loss of lignin and cellulose was determined. The average of three identical test samples from each group was calculated.

**TABLE 1:** Factor-level table of the orthogonal test.

Level	Factors			
	A	B	C	D
	Temperature (°C)	pH	Culture time (d)	Solid-liquid ratio
1	28	5	30	1:1.5
2	30	6	40	1:2.5
3	32	7	50	1:3.5

### 2.6 Analyses of bamboo composition

Untreated and pretreated bamboo sample chemical composition was analyzed based on the standard protocols developed by the National Renewable Energy Laboratory (NREL) (Sluiter et al., 2008). All analyses were performed in triplicate.

Weight loss values for different bamboo components were calculated by comparing initial and final weights in pretreated samples as follows:

$$Weight\;loss\left(\%\right)=\frac{\left(W1-W2\right)}{W1}\times 100$$ (1)

where W1 and W2 respectively represent the sample weight prior to and after pretreatment.

Selective lignin degradation is a key feature of lignin-degrading fungi. For this study, this trait was quantified using selectivity values based on the differences in the proportional loss of lignin and cellulose content (Shirkavand et al., 2017):

$$Selectivity\;value=\frac{Lignin\;loss\;at\;the\;time\;of\;pretreatment}{Cellulose\;loss\;at\;the\;time\;of\;pretreatment}$$ (2)

### 2.7 Physical and chemical characterization of pretreated bamboo samples

Untreated and fungally pretreated bamboo powder samples were subjected to FTIR analyses with an FTIR-650 instrument. Briefly, 2 mg of sample and 200 mg of KBr (1:100 weight ratio) were combined before analysis, and FTIR spectra were analyzed in the 500–4,000 cm^−1^ at a resolution of 4 cm^−1^. An Ultima IV instrument was used for XRD measurements with the following settings: 40 kV and 40 mA. Samples were characterized using a scanning angle of 5°–40° (2θ), with a scanning speed of 8°/min. Changes in the microstructural characteristics of bamboo samples following pretreatment were assessed with a scanning electron microscope (SEM, Hitachi TM 3000, Japan) at an accelerating voltage of 15–20 kV. With regard to the peak height method, the crystallinity index (CrI) was calculated based on the equation below (Li et al., 2017):

$$CrI=\frac{I_{002}-I_{am}}{I_{002}}\times 100$$ (3)

Where I_002_ is the intensity of diffraction from the 002 plane at 2θ = 22.2°, and I_am_ is the intensity of background scatter measured at 2θ = 18.3°.

## 3 Results and discussion

### 3.1 Analyses of enzymatic activity

**FIGURE 1:**
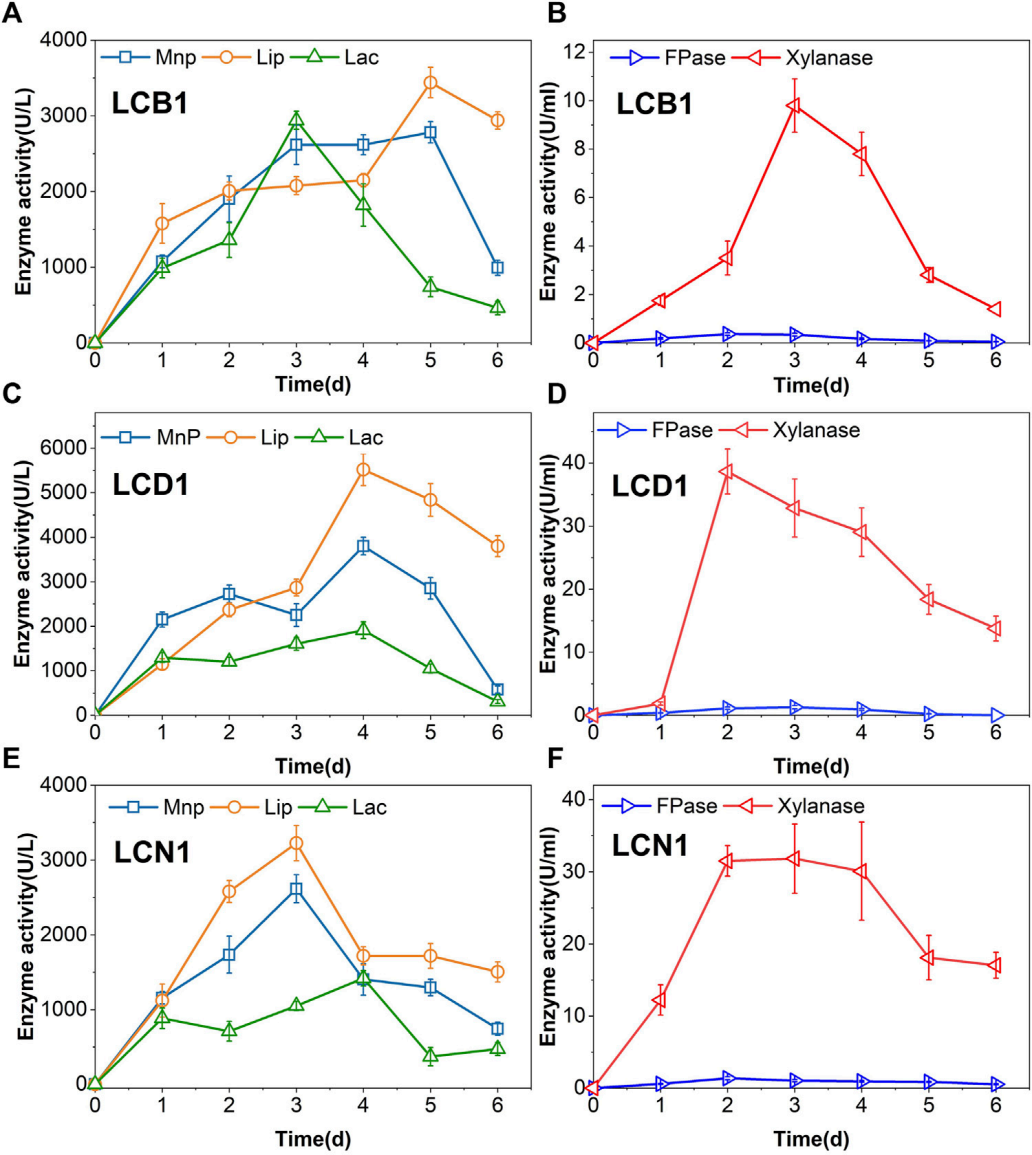
Fungal enzymatic activity levels over the incubation period. **(A,B)** LCB1, **(C,D)** LCD1, **(E,F)** LCN1.

Fungi and bacteria primarily break down lignin by secreting enzymes into the extracellular space, with the activity of these enzymes, including Lac, LiP, and MnP, ultimately governing lignin degradation. Lac activity levels rose steadily during the first 3 days of LCB1 incubation after which they declined, whereas peak LiP and MnP activity levels were evident on day 5 (Figure 1A), with maximum respective Lac, LiP, and MnP levels of 2,941, 3,441, and 2,782 U/L.

For LCB1 fungal cultures, maximal FPase activity levels of 0.37 U/mL were evident on day 2, whereafter these levels declined gradually to a minimum of 0.05 U/mL on day 6 (Figure 1B). Xylanase is the primary enzyme responsible for hemicellulose degradation through its ability to break xylan down into xylose and xylooligosaccharide. Peak xylanase activity levels (9.8 U/mL) were evident on day 3 of culture, where after they gradually declined. In LCD1 and LCN1 fungal cultures, both FPase and xylanase activity levels showed the same trend as LCB1 (Figures 1D, F). Notably, all these three strains exhibited significantly lower FPase activity levels relative to xylanase activity, suggesting that these fungi preferentially degrade hemicellulose rather than cellulose. Hemicellulose degradation disrupts lignin-carbohydrate complex linkages, resulting in greater lignin exposure (Qi et al., 2022), thus enabling more robust lignin biodegradation over time.

LCD1 exhibited distinct enzymatic kinetics, with MnP and LiP activity levels peaking at 3,801 and 5,519 U/L, respectively, on day 4, while Lac activity levels remained lower than those for these other two enzymes throughout the incubation period. Lac enzymatic activity peaked at 1,912 U/L on day 4 (Figure 1C). MnP and LiP activity levels of LCN1 peaked at 2,617 and 3,226 U/L, respectively, on day 3. Laccase levels were also lower than those of the other two lignin-degrading enzymes (Figure 1E). High levels of activity for these lignin-degrading enzymes were evident in the present study.

Prior studies have found that few fungi are capable of producing all three of these enzymes, with most secreting just one or two (Xu et al., 2018). In contrast, the present results demonstrate that the three strains are capable of simultaneously secreting all three of these enzymes such that high activity levels were observed, potentially contributing to an improved capacity for lignin removal.

### 3.2 Optimization of fungi culture conditions

The results of our exploration of the three fungi with different co-cultivation combinations are shown in Table 2. The lignin weight loss by the two or three strains co-cultured was significantly higher than that of any single strain culture. It may be possible that co-cultivation of interacting fungi over-expresses lignolytic enzymes during pretreatment process giving synergistic and combinatorial effect for efficient delignification (Meehnian et al., 2017). The highest lignin weight loss was observed in LCB1+LCN1 combination. It was then decided to use LCB1+LCN1 combination for further tests.

**TABLE 2:** The effect of different co-cultivation combination of three fungi on the weight loss of cell wall components after the 30-day pretreatment period.

Strain	Lignin weight loss (%)	Hemicellulose weight loss (%)	Cellulose weight loss (%)	Cellulose retention ratio (%)
LCB1	35.52	39.55	11.22	88.78
LCD1	30.41	27.53	9.56	90.44
LCN1	23.65	55.69	4.32	95.68
LCB1+LCD1	47.57	43.23	10.23	89.77
LCB1+LCN1	67.36	60.12	5.12	94.88
LCD1+LCN1	63.59	52.36	8.46	91.54
LCB1+LCD1+LCN1	60.26	47.38	12.12	87.88

Culture temperature 28°C, pH 7, solid-liquid ratio 1:2.5.

The sovereign weights (0.7, 0.3) of the two indexes were determined using the expert experience approach, and a comprehensive score was then produced. According to Table 3, the lignin weight loss, cellulose retention ratio, and comprehensive scores are affected by primary and secondary factors in the following order: D > A > C > B, A > B > C > D, and A > B > D > C, respectively. The A1B1C2D2 and A2B1C2C2 combinations were tried three times to confirm the ideal combination. According to the verification test’s findings, when the degradation time was 40 days in the combination of A2B1C2C2, the weight loss of lignin was 76.37%, the cellulose retention ratio was 94.73%, and the comprehensive score was 0.99, so the optimum level is A2 (30°C) B1 (5) C2 (40 d) D2 (1:2.5). Previous studies evaluated different fungal strains for pretreatment of bamboo and 13%–53% lignin weight losses, 8.8%–26.2% hemicellulose weight loss and 20.3% cellulose weight loss were observed, respectively (Suhara et al., 2012; Zeng et al., 2012; Ma et al., 2017; Liu et al., 2023). Compared with these data, co-cultivation of LCB1 and LCN1 in our study showed a higher delignification rate for bamboo. Meanwhile, cellulose was conserved by the process of pretreatment, indicating that co-cultivation of LCB1 and LCN1 was an ideal pretreatment method for lignin depletion and the efficient utilization of cellulose in bamboo.

**TABLE 3:** Results of orthography test and range analysis.

Test number	Experimental factor				Weight loss of lignin	Cellulose retention ratio	Comprehensive scores
	A	B	C	D			
1	1	1	1	1	58.56	93.76	0.86
2	1	2	2	2	69.32	89.56	0.97
3	1	3	3	3	52.13	91.26	0.80
4	2	1	2	3	65.38	94.35	0.95
5	2	2	3	1	50.65	91.29	0.79
6	2	3	1	2	61.89	97.36	0.92
7	3	1	3	2	59.36	94.26	0.89
8	3	2	1	3	45.36	93.63	0.75
9	3	3	2	1	49.74	93.87	0.79
Analysis of lignin weight loss							
k1	60.00	61.10	55.27	52.98			
k2	59.31	55.11	61.48	63.52			
k3	51.49	54.59	54.04	54.29			
R	8.51	6.51	7.44	10.54			
Better level	A1	B1	C2	D2			
Primary and secondary factors D > A > C > B							
Analysis of cellulose retention ratio							
k1	91.52	94.12	94.91	92.97			
k2	94.33	91.49	92.59	93.73			
k3	93.92	94.16	92.27	93.08			
R	2.81	2.67	2.64	0.76			
Better level	A2	B3	C1	D2			
Primary and secondary factors A > B > C > D							
Analysis of comprehensive scores							
k1	0.88	0.90	0.84	0.81			
k2	0.89	0.84	0.90	0.93			
k3	0.81	0.84	0.83	0.83			
R	0.08	0.06	0.07	0.12			
Better level	A2	B1	C2	D2			
Primary and secondary factors A > B > D > C							
Verication test							
A1B1C2D2					72.33	92.43	0.98
A2B1C2D2					76.37	94.73	0.99

### 3.3 Analysis of the chemical composition of pretreated bamboo

**FIGURE 2:**
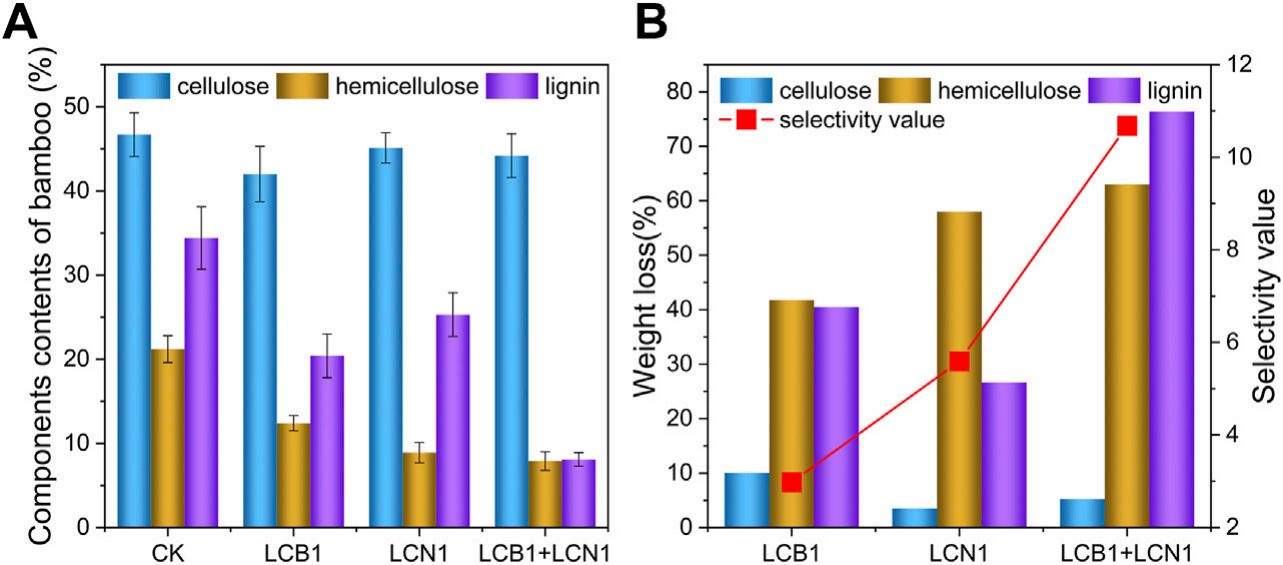
**(A)** Analysis of the composition of untreated and pretreated bamboo samples after pretreatment under optimum condition. **(B)** Lignin, hemicellulose, and cellulose weight loss and the degree of selectivity, as measured with the selectivity value, after pretreatment under optimum condition.

To more fully explore the impact of bio-pretreatment on bamboo composition, the NREL method was used to quantify lignin, cellulose, and hemicellulose levels therein (Figure 2A). Following a 40-day pretreatment period under optimum conditions, the relative cellulose and hemicellulose content in these samples declined from 46.74% and 21.24% to 42.02% and 12.36% and to 45.06% and 8.93% following LCB1 and LCN1 pretreatment, respectively. Cellulose levels did not decline significantly following pretreatment with an LCB1 and LCN1 co-culture, falling from 46.74% to 44.24%. Hemicellulose content decreased significantly in all three groups despite the preservation of cellulose content (Figure 2B), in line with the enzymatic activity assay results demonstrating low levels of cellulase activity together with high levels of hemicellulase activity.

Following pretreatment with the LCB1 and LCN1 strains, the relative lignin content in these bamboo samples declined from 34.41% to 20.48% and 25.25%, respectively, emphasizing the lignin-degrading activity of these two strains. Strikingly, LCB1 and LCN1 co-culture facilitated a rapid drop in lignin content from 34.41% to 8.13%. Similar levels of lignin degradation have previously been reported when corn stover was pretreated using a crude lignocellulolytic enzyme solution generated by *Coprinus comatus* and *Trichoderma reesei* (Ma and Ruan, 2015).

Over a 40-day biodegradation period, both the LCB1 and LCN1 strains facilitated significant 40.48% and 26.62% reductions in lignin weight, respectively, while this lignin weight loss rose to 76.37% for the co-culture group (Figure 2B). These results emphasize the ability of these fungi to selectively degrade lignin, particularly when co-cultured. This selective breakdown of lignin resulted in the conservation of cellulose content during the pretreatment period such that it can be converted into glucose through subsequent processing. LCB1 and LCN1 co-culture pretreatment thus represents an ideal approach to lignin depletion conducive to the more efficient utilization of bamboo-derived cellulose.

### 3.4 Physical and chemical characterization of pretreated bamboo culm samples

**FIGURE 3:**
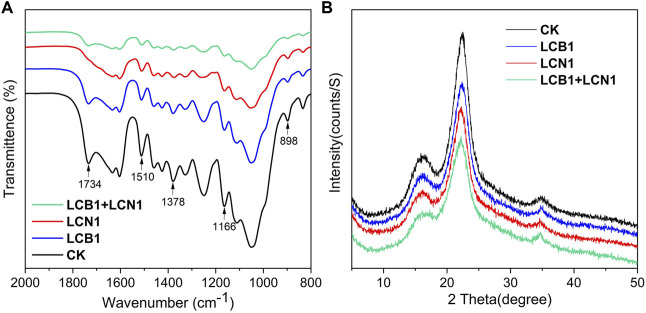
**(A,B)** FTIR **(A)** and XRD **(B)** spectra for untreated and pretreated bamboo culms.

FTIR analyses were next conducted as a means of exploring the pretreatment-related chemical changes in bamboo samples (Figure 3A). Proportional reductions in lignin peak intensity (1,510 cm^−1^) were evident in LCB1- and LCN1-pretreated bamboo samples after the 40-day pretreatment period, with this peak having been assigned to the aromatic skeleton of lignin (Zhang et al., 2007). Even more pronounced reductions in the 1,510 cm^−1^ peak were evident in samples pretreated with LCB1+LCN1, emphasizing the robust lignin-degrading activity associated with the co-culture of these two fungi.

To better assess the selective degradation of lignin in these bamboo samples, detailed FTIR spectroscopic analyses of these samples were performed as reported previously (Pandeya and Pitmanb, 2003). The relative changes in lignin peak intensity (1,510 cm^−1^) as compared to the peak areas for other carbohydrate peaks (1,734, 1,378, 1,166, and 898 cm^−1^) are shown in Table 4. These four carbohydrate peaks were respectively assigned to unconjugated C=O in xylans, C–H deformation in cellulose and hemicellulose, C–O–C vibrations in cellulose and hemicellulose, and C–H deformation in cellulose (Zhang et al., 2007). Significant reductions in the lignin/carbohydrate ratio were observed in LCB1, LCN1, and LCB1+LCN1 pretreated bamboo samples after 40 days, with the exception of the *I*_*1510*_ */I*_*1734*_ ratio. These results reaffirmed the selective ability of these fungi to degrade lignin. The increase in the *I*_*1510*_ */I*_*1734*_ ratio was primarily attributable to hemicellulose degradation.

**TABLE 4:** Ratios of the intensity of the lignin-associated band relative to other carbohydrate bands in pretreated bamboo samples.

	Relative intensities of aromatic skeletal vibration (*I*_*1510*_) against typical bands for carbohydrates			
Group	*I*_*1510*_ */I*_*1734*_	*I*_*1510*_ */I*_*1378*_	*I*_*1510*_ */I*_*1166*_	*I*_*1510*_ */I*_*898*_
CK	1.53	1.48	1.87	3.67
LCB1	1.48	1.33	1.63	3.35
LCN1	5.45	1.28	1.56	2.76
LCB1+LCN1	3.76	1.25	1.35	2.14

The crystallinity of these bamboo samples was also assessed via XRD spectroscopy. A significant decrease in the diffraction peak intensity at 22.2°, which is characteristic of the cellulose crystalline phase in bamboo fibers (Saikia et al., 2015), was observed following fungal pretreatment (Figure 3B). This suggests that fungal pretreatment significantly altered bamboo culm crystallinity. As shown in Table 5, the reduction in CrI makes the pretreated bamboo more suitable for further bioprocessing. Enhanced microbial and enzymatic accessibility to the substrate shall be possible due to cellulose crystallinity reduction.

**TABLE 5:** Comparison of CrI values of untreated and pretreated bamboo.

Sample	I_002_	I_am_	CrI
CK	4,501	1,844	59.03
LCB1	3,568	1,597	55.24
LCN1	3,064	1,341	56.23
LCB1+LCN1	2,484	1,185	52.29

**FIGURE 4:**
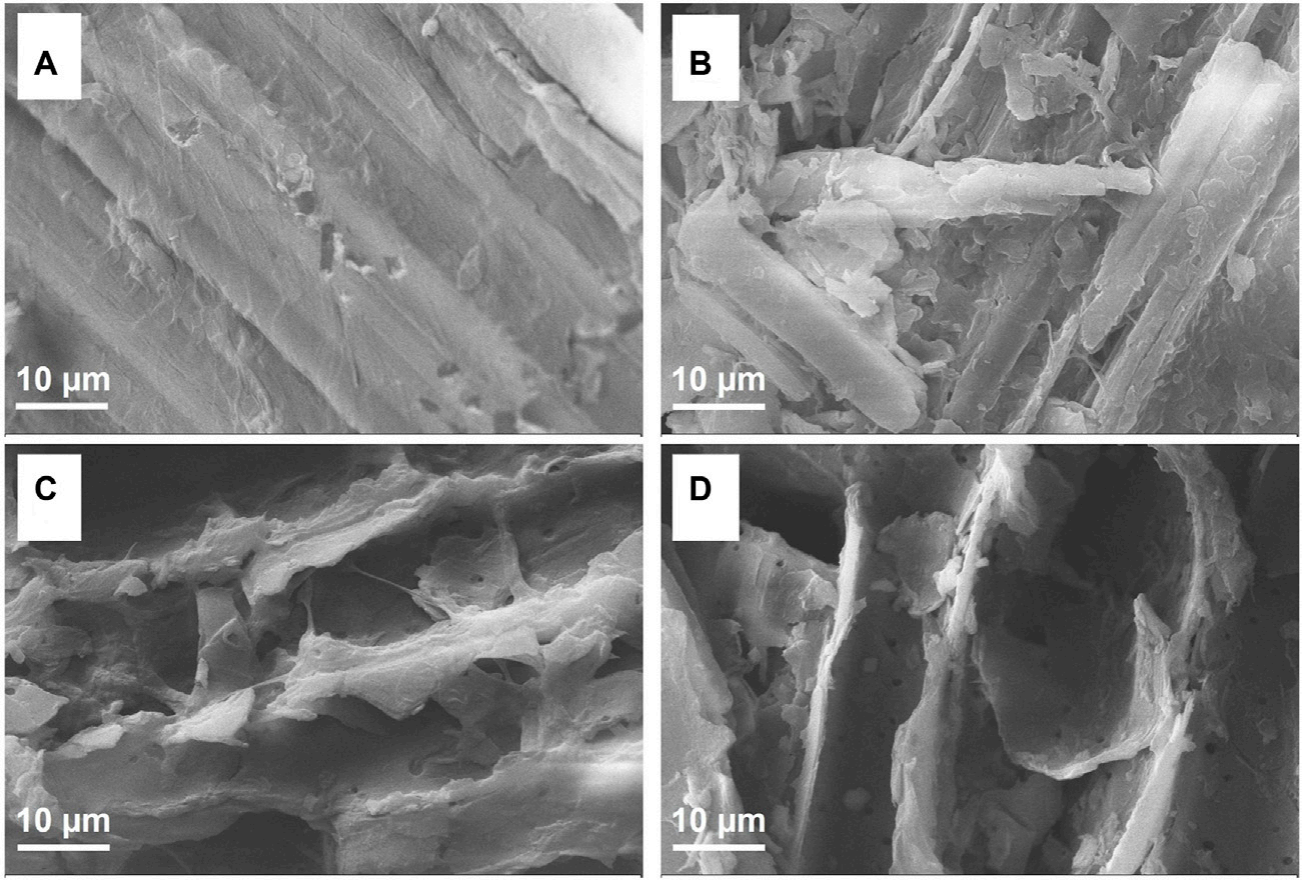
SEM images of bamboo samples that were **(A)** untreated or pretreated for 40 days with **(B)** LCB1, **(C)** LCN1, or **(D)** LCB1+LCN1.

The microstructural properties of untreated and fungally pretreated bamboo samples are presented in Figure 4, revealing pronounced structural differences. The surface of untreated bamboo samples was smooth and compact, with naturally stretched, well-ordered fiber bundles consistent with a rigid structure (Figure 4A). In contrast, the pretreated bamboo samples exhibited markedly reduced surface integrity, with pitting and holes being clearly evident following LCB1- or LCN1-mediated processing (Figures 4B, C). These changes were even more pronounced for samples that were subjected to co-culture pretreatment such that fibers were highly disordered and damaged, resulting in a rougher and more fragmented surface (Figure 4D).

## 4 Conclusion

In summary, the newly isolated LCB1 and LCN1 fungal strains exhibit rapid ligninolytic enzyme production conducive to a shorter fermentation interval. The physicochemical characterization of bamboo samples processed with these fungi revealed that the LCB1 and LCN1 co-culture pretreatment of bamboo culms more effectively depleted lignin than pretreatment with either of these fungi in isolation. Importantly, co-culture pretreatment with these fungi primarily resulted in the degradation of lignin and hemicellulose while selectively leaving the cellulose content intact for further downstream processing.

## Data Availability

The raw data supporting the conclusion of this article will be made available by the authors, without undue reservation.
