# Supplementary material for: The use of newly isolated fungal cultures for the selective delignification of bamboo culms
Source: Front Bioeng Biotechnol. 2023 Aug 31;11:1265420. doi: 10.3389/fbioe.2023.1265420 (PMC10501718; doi:10.3389/fbioe.2023.1265420)
Supplement: Supplementary file 1 [file DataSheet1.doc]

**Supplementary Information**

**The Use of Newly Isolated Fungal Cultures for the Selective Delignification of Bamboo Culms**

Bo Zhao^a,1^, Rui Liu ^a,1^, Qi Guo^a^, Gang Xu^a^, Li Zhang^b^, Peng Sun^b^, Ying Cao^a*^, Shanglian Hu^a*^

^a^ School of Life Science and Engineering, Bamboo Research Institute, Southwest University of Science and Technology, Mianyang 621010, China

^b^ Sichuan Academy of Forestry, Chengdu 610081, China

* Correspondence to: Prof. Shanglian Hu (E-mail: [hnw57@ustc.edu.cn](mailto:hnw57@ustc.edu.cn)) and Prof Ying Cao (E-mail: xkd118@163.com), School of Life Science and Engineering, Southwest University of Science and Technology, 59# Qinglong road, Mianyang, 621010, People’s Republic of China

^1^These authors contribute equally to this work.

Supporting Information Includes:2 figures and 1 table

**Table S1. Details of location where the fungi were collected.**

| Locality | Fungal isolate |
| --- | --- |
|  | *Paracremonium sp.* LCB1, *Clonostachys compactiuscula* LCD1 and *Clonostachys compactiuscula* LCN1 |
| Isolation source | Decayed bamboo |
| Host | *Dendrocalamus latiflorus* Munro |
| Locality | Longchang, Sichuan Provence, China |
| GPS coordinates | 105.315078, 29.459384 |
| Altitude | 340 m |
| Season | Spring |
| Collection date | 7 March 2022 |
| Daily temperature range on the day of sample collection | 9-25 °C |


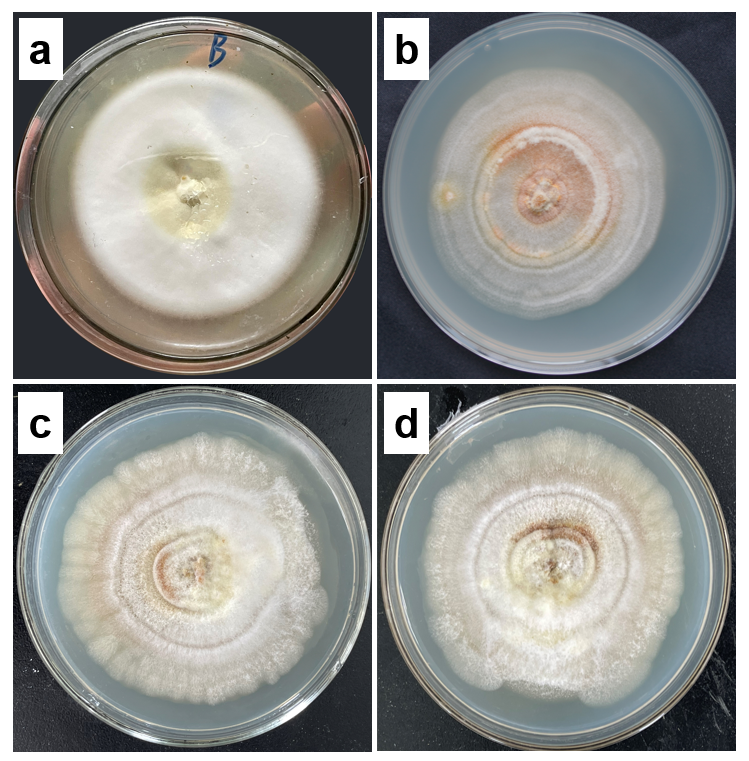


**Fig. S1.** Fungi stains grown in PDA plates and incubated at 28 ℃ for 7 days. (a)LCB1, (b)LCD1, (c)LCN1, (d)LCB1+LCN1


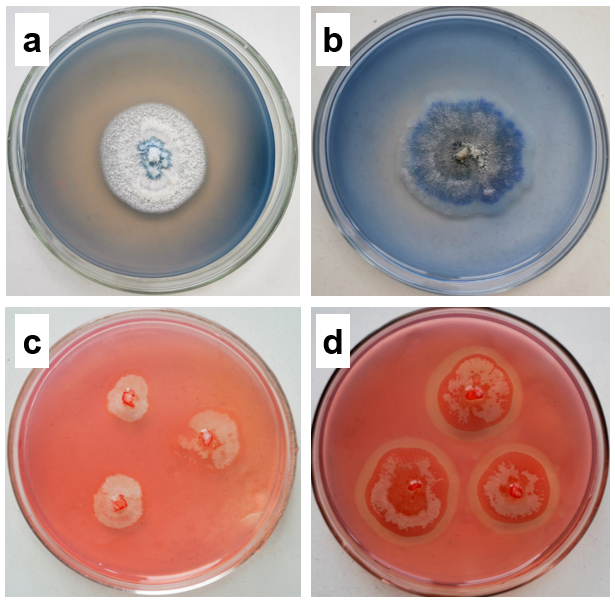


**Fig. S2.** Screening of potential selective lignin-degrading fungal isolate on PDA plate. (a) LCB1+ aniline blue, (b) LCN1+ aniline blue, (c) LCB1+Congo red, (d) LCN1+Congo red
